# First-line cetuximab improves the efficacy of subsequent bevacizumab for RAS wild-type left-sided metastatic colorectal cancer: an observational retrospective study

**DOI:** 10.1038/s41598-020-69230-5

**Published:** 2020-07-23

**Authors:** Shousheng Liu, Chang Jiang, Lin Yang, Jinsheng Huang, Roujun Peng, Xiaopai Wang, Wenzhuo He, Long Bai, Yixin Zhou, Bei Zhang, Liangping Xia

**Affiliations:** 1 State Key Laboratory of Oncology in South China, Collaborative Innovation Centre for Cancer Medicine, Sun Yat-sen University Cancer Center, NO. 651 Dongfeng East Road, Guangzhou, 510060 People’s Republic of China; 2 Department of the General Medicine, Sun Yat-sen University Cancer Center, NO. 651 Dongfeng East Road, Guangzhou, 510060 People’s Republic of China; 3 Department of Radiation Oncology, Nanfang Hospital, Southern Medical University, NO. 1838 Guangzhou Avenue North, Guangzhou, 510515 People’s Republic of China; 4 Department of Pathology, Guangzhou First People’s Hospital, Guangzhou Medical University, The Second Affiliated Hospital, South China University of Technology, NO. 1 Panfu Road, Guangzhou, 510080 People’s Republic of China

**Keywords:** Diseases, Gastroenterology, Medical research, Oncology

## Abstract

The optimal targeted therapy sequence in patients of RAS wild-type left-sided metastatic colorectal cancer (mCRC) remains controversial, and few studies focus on the impact of first-line targeted agents on second-line ones. We enrolled 101 left-sided mCRC patients with RAS wild-type status, of which 50 cases received bevacizumab plus chemotherapy in both first-line and second-line therapies (Group A) and 51 cases received first-line cetuximab plus chemotherapy followed by second-line bevacizumab-containing regimens (Group B). The progression free survival (PFS) and overall survival (OS) from start of first-line (PFS 1nd and OS 1nd) and second-line (PFS 2nd and OS 2nd) therapy were compared between the two groups. PFS 1nd was comparable (10.0 vs 10.4 months; *p* = 0.402), while PFS 2nd (4.6 vs 7.9 months; *p* = 0.002), OS 1nd (26.8 vs 40.0 months; *p* = 0.011), and OS 2nd (15.2 vs 22.3 months; *p* = 0.006) were all poorer in group A compared with group B. Our study in combination with previous clinical data suggest that first-line application of cetuximab may provide a favorable condition for promoting the effect of subsequent bevacizumab, thus representing the optimal targeted therapy sequence in patients of RAS wild-type left-sided mCRC.

## Introduction

The current classic chemotherapy schemes to treat metastatic colorectal cancer (mCRC) include: combinations of fluorouracil and oxaliplatin (FOLFOX); fluorouracil and irinotecan (FOLFIRI); capecitabine and oxaliplatin (XELOX); and fluorouracil plus oxaliplatin and irinotecan (FOLFOXIRI)^1–3^. The introduction of epidermal growth factor receptor (EGFR) inhibitors such as cetuximab and vascular endothelial growth factor (VEGF) inhibitors such as bevacizumab into combination with chemotherapy further improves the efficacy, and thus has become the standard therapeutic schedule for mCRC^4–6^. There is growing evidence that EGFR inhibitors confer little benefit to patients with mCRC if the primary tumor located on the right side (caecum to transverse colon) instead of left side (splenic flexure to rectum)^7,8^, while VEGF inhibitors exert a similar effect on left- and right-sided tumors. Therefore, VEGF inhibitors rather than EGFR inhibitors are recommended in the treatment of right-sided mCRC.

When it comes to left-sided mCRC, which kind of targeted biologic agents is preferred in first-line therapy? Several clinical trials recommended EGFR inhibitors-containing regimens because it resulted in better clinical outcomes compared with VEGF inhibitors-containing regimens^9,10^; However, some other studies found no statistic differences in overall survival (OS) between the two biological agents^11^. Besides, VEGF inhibitors are more cost effective than EGFR inhibitors in first-line therapy for mCRC in the perspective of economics^12,13^. Therefore, the addition of EGFR or VEGF inhibitors to chemotherapy are equivalently recommended in the first-line therapy for RAS wild-type left-sided mCRC patients in NCCN Guidelines (Version 2.2018). One aim of our study was to validate the preferred first-line biological agent choice of RAS wild-type left-sided mCRC in real-world community settings based on the data from our center.

More importantly, most of the previous studies about selection of targeted therapy mainly focused on first-line therapy, and there are very little researches concerning second-line therapy through it also plays an important role in clinical outcomes. Recently, several clinical trials support the continuation of bevacizumab crossover instead of converting to anti-EGFR agents for mCRC patients with wild-type RAS that progressed with first-line bevacizumab plus chemotherapy, because the former strategy produced better OS and progression free survival (PFS) results though with a nonsignificant difference^14–16^, revealing that first-line targeted agents might exert influence on the later-line targeted therapy.

In this retrospective study, we focused on RAS wild-type left-sided mCRC patients with different first-line whereas the same second-line biologic agents to investigate the influence of anti-EGFR/VEGF antibodies on anti-VEGF agent, as well as provide some evidence for the appropriate treatment sequence in this particular group of patients.

## Results

### Patients characteristics

A total of 101 patients were enrolled in the study, including 50 patients receiving bevacizumab-containing regimens in both first-line and second-line therapies (Group A) and 51 cases receiving first-line cetuximab-containing regimens followed by bevacizumab-containing regimens in second-line therapy (Group B). The clinical baseline features of the 101 patients by treatment groups are shown in Table 1. The cut-off values of age and CEA level were calculated from the medians. Primary tumor resection was conducted in 19 (38.0%)patients from group A and 26 (51.0%) patients from group B, and metastatic lesions resection was conducted in 15 (30.0%) patients from group A and 17 (33.3%) patients from group B at any time during stage IV. Oxaliplatin-based regimens (FOLFOX or XELOX) were implemented in 36 (72.0%) patients from group A and 28 (54.9%) patients from group B, while irinotecan-based regimen (FOLFIRI) was implemented in 12 (24.0%) patients from group A and 20 (39.2%) patients from group B in first-line therapy. The information of third-line treatment can be found as Supplementary information Table S1 online. All factors were balanced between the two groups in statistics (all *p* > 0.05). The median follow-up time in group A and group B was 50.36 months and 47.31 months respectively.

**Table 1:** Clinicopathological characteristics of mCRC patients based on treatment groups.

Characteristics	Treatment group A	Treatment group B	*p* value
**Number of cases (n, %)**			
**Age at diagnosis as stage IV (years)**			0.371
< 52	27 (54.0)	23 (45.1)	
≥ 52	23 (46.0)	28 (54.9)	
**Gender**			0.891
Male	33 (66.0)	33 (64.7)	
Female	17 (34.0)	18 (35.3)	
**WHO PS**			0.583
0–1	39 (78.0)	42 (82.4)	
≥ 2	11 (22.0)	9 (17.6)	
**Tumor histological grade**			0.109
Well-differentiated	3 (6.0)	1 (2.0)	
Moderately differentiated	30 (60.0)	30 (58.8)	
Poorly differentiated	10 (20.0)	18 (35.3)	
Mucinous	7 (14.0)	2 (3.9)	
**Localization of the primary tumor**			0.490
Colon	26 (52.0)	30 (58.8)	
Rectum	24 (48.0)	21 (41.2)	
**Number of metastatic sites**			0.362
1	20 (40.0)	25 (49.0)	
> 1	30 (60.0)	26 (51.0)	
**CEA**			0.307
< 19.99	27 (55.1)	21 (44.7)	
≥ 19.99	22 (44.9)	26 (55.3)	
**Primary tumor resection**			0.189
Yes	19 (38.0)	26 (51.0)	
No	31 (62.0)	25 (49.0)	
**Metastases resection**			0.719
Yes	15 (30.0)	17 (33.3)	
No	35 (70.0)	34 (66.7)	
**Chemotherapy used in first-line**			0.203
Oxaliplatin-based	36 (72.0)	28 (54.9)	
Irinotecan-based	12 (24.0)	20 (39.2)	
Others	2 (4.0)	3 (5.9)	
**Chemotherapy used in second-line**			0.144
Oxaliplatin-based	20 (40.0)	25 (49.0)	
Irinotecan-based	28 (56.0)	20 (39.2)	
Others	2 (4.0)	6 (11.8)	

Treatment group A: bevacizumab-containing regimens in both first-line and second-line therapies.

Treatment group B: first-line cetuximab-containing regimens followed by second-line bevacizumab-containing regimens.

### Response rates in group A and group B

Response parameters are listed in Table 2. During first-line therapy, 22 (44.0%) patients in group A and 33 (64.7%) patients in group B achieved partial response, 26 (52.0%) patients in group A and 13 (25.5%) patients in group B achieved stable disease. Therefore, the first-line ORR in group A was lower than that in group B (44.0% vs 64.7%, *p* = 0.037), while first-line DCR was comparable between two groups (96.0% vs 90.2%, *p* = 0.251). During second-line therapy, ORR was 16.0% in group A and 27.5% in group B (*p* = 0.163), while DCR was 64.0% in group A and 82.4% in group B (*p* = 0.037), respectively.

**Table 2:** Response rate of mCRC patients in two treatment groups.

Parameters	Treatment group A	Treatment group B	*p* value
**Evaluable response to first-line therapy (n, %)**			
CR	0 (0)	0 (0)	
PR	22 (44.0)	33 (64.7)	
SD	26 (52.0)	13 (25.5)	
PD	2 (4.0)	5 (9.8)	
ORR	44.0%	64.7%	**0.037**
DCR	96.0%	90.2%	0.251
**Evaluable response to second-line therapy (n, %)**			
CR	0 (0)	0 (0)	
PR	8 (16.0)	14 (27.5)	
SD	24 (48.0)	28 (54.9)	
PD	18 (36.0)	9 (17.6)	
ORR	16.0%	27.5%	0.163
DCR	64.0%	82.4%	**0.037**

Treatment group A: bevacizumab-containing regimens in both first-line and second-line therapies.

Treatment group B: first-line cetuximab-containing regimens followed by second-line bevacizumab-containing regimens.

Bold values indicate significant differences between two groups.

*CR* complete response, *PR* partial response, *SD* stable disease, *PD* progression of disease, *ORR* overall response rate, *DCR* disease control rate.

### PFS 1nd and PFS 2nd in group A and group B

**Figure 1:**
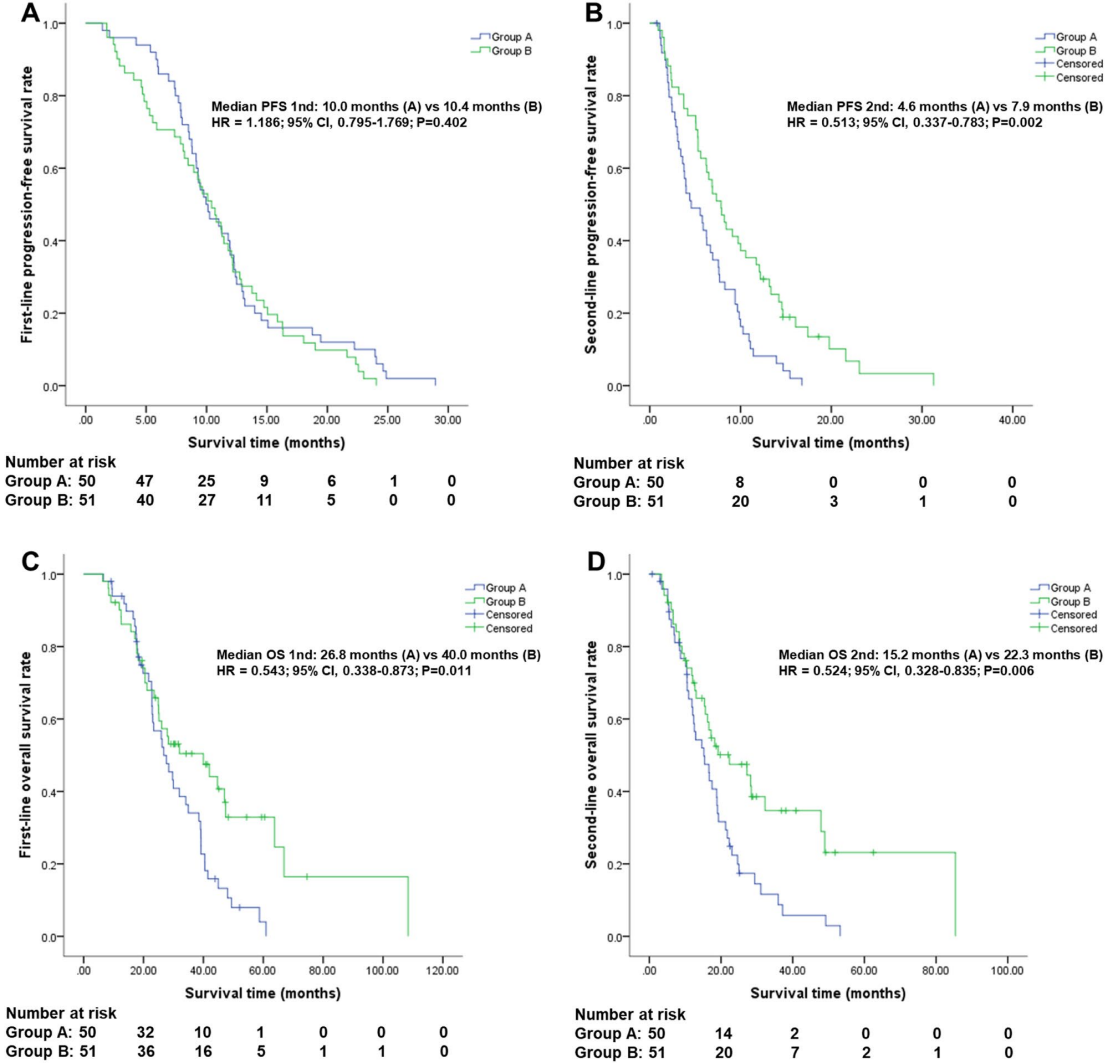
PFS and OS comparison between group A and group B using Kaplan–Meier method. Group A: bevacizumab-containing regimens in both first-line and second-line therapies; Group B: first-line cetuximab-containing regimens followed by second-line bevacizumab-containing regimens. (**A**) First-line PFS: from the beginning of first-line therapy to first disease progression; (**B**) Second-line PFS: from the date when second-line therapy started to second progression in disease; (**C**) First-line OS: from first application of first-line therapy to death resulting from mCRC; (**D**) Second-line OS: from beginning of second-line therapy to death resulting from mCRC. The difference was significant if *p* < 0.05 by log-rank test.

As shown in Fig. 1A,B, patients in group B had a comparable PFS 1nd (hazard ratio [HR] = 1.186; 95% CI, 0.795–1.769; *p* = 0.402) and better PFS 2nd (HR = 0.513; 95% CI, 0.337–0.783; *p* = 0.002) compared with patients in group A. Median PFS 1nd was 10.0 months (95% CI, 8.0–11.9 months) in group A and 10.4 months (95% CI, 8.5–12.4 months) in group B. Median PFS 2nd was 4.6 months (95% CI, 2.1–7.0 months) in group A and 7.9 months (95% CI, 5.9–9.8 months) in group B.

Univariate analysis indicated that tumor histological grade, number of metastatic sites and metastases resection were significantly associated with PFS 1nd, while metastases resection, second-line chemotherapy regimens and treatment group were significantly associated with PFS 2nd (Tables 2, 3). When it came to the multivariate analysis, none of the above meaningful factors had statistical correlation with PFS 1nd and only metastases resection and group B remained independently associated with a better PFS 2nd (Tables 3, 4).

**Table 3:** Univariate and multivariate analysis for factors associated with PFS 1nd.

Variables	Univariate analysis			Multivariate analysis		
	HR	95%CI	*p* value	HR	95%CI	*p* value
**Age at diagnosis as stage IV (years)**						
< 52	1					
≥ 52	0.905	(0.608–1.346)	0.622			
**Gender**						
Male	1					
Female	0.918	(0.606–1.391)	0.687			
**WHO PS**						
0–1	1					
≥ 2	1.140	(0.697–1.866)	0.601			
Tumor histological grade			**0.030**			0.389
**Localization of the primary tumor**						
Colon	1					
Rectum	0.757	(0.505–1.134)	0.176			
**Number of metastatic sites**						
1	1			1		
> 1	1.768	(1.174–2.662)	**0.006**	1.580	(0.986–2.449)	0.058
**CEA**						
< 19.99	1					
≥ 19.99	0.943	(0.628–1.415)	0.777			
**Primary tumor resection**						
Yes	1					
No	0.835	(0.557–1.249)	0.380			
**Metastases resection**						
Yes	1			1		
No	1.627	(1.061–2.494)	**0.026**	1.502	(0.965–2.338)	0.071
**Chemotherapy used in first-line**			0.682			
Oxaliplatin-based	1					
Irinotecan-based	0.845	(0.548–1.302)	0.445			
Others	1.156	(0.463–2.883)	0.757			
**Treatment group**						
Group A	1					
Group B	1.186	(0.795–1.769)	0.404			

Treatment group A: bevacizumab-containing regimens in both first-line and second-line therapies.

Treatment group B: first-line cetuximab-containing regimens followed by second-line bevacizumab-containing regimens.

Bold values indicate significant differences between two groups.

**Table 4:** Univariate and multivariate analysis for factors associated with PFS 2nd.

Variables	Univariate analysis			Multivariate analysis		
	HR	95%CI	*p* value	HR	95%CI	*p* value
**Age at diagnosis as stage IV (years)**						
< 52	1					
≥ 52	0.856	(0.565–1.295)	0.461			
**Gender**						
Male	1					
Female	1.222	(0.801–1.864)	0.353			
**WHO PS**						
0–1	1					
≥ 2	1.005	(0.954–1.058)	0.859			
Tumor histological grade			0.105			
**Localization of the primary tumor**						
Colon	1					
Rectum	0.767	(0.511–1.153)	0.202			
**Number of metastatic sites**						
1	1					
> 1	1.421	(0.932–2.166)	0.102			
**CEA**						
< 19.99	1					
≥ 19.99	0.915	(0.605–1.384)	0.674			
**Primary tumor resection**						
Yes	1					
No	1.080	(0.718–1.625)	0.711			
**Metastases resection**						
Yes	1			1		
No	1.837	(1.171–2.882)	**0.008**	1.784	(1.134–2.807)	**0.012**
**Chemotherapy used in first-line**			0.807			
Oxaliplatin-based	1					
Irinotecan-based	0.866	(0.559–1.342)	0.520			
Others	0.900	(0.359–2.255)	0.822			
**Chemotherapy used in second-line**			**0.067**			0.139
Oxaliplatin-based	1			1		
Irinotecan-based	1.440	(0.946–2.192)	0.089	1.443	(0.945–2.203)	0.090
Others	0.572	(0.224–1.460)	0.243	0.726	(0.279–1.894)	0.513
**Treatment group**						
Group A	1			1		
Group B	0.513	(0.337–0.783)	**0.002**	0.560	(0.364–0.862)	**0.008**

Treatment group A: bevacizumab-containing regimens in both first-line and second-line therapies.

Treatment group B: first-line cetuximab-containing regimens followed by second-line bevacizumab-containing regimens.

Bold values indicate significant differences between two groups.

### OS 1nd and OS 2nd in group A and group B

As shown in Fig. 1C,D, patients in group B had a better OS 1nd (HR = 0.543; 95% CI, 0.338–0.873; *p* = 0.011) and OS 2nd (HR = 0.524; 95% CI, 0.328–0.835; *p* = 0.006) compared with patients in group A. Median OS 1nd was 26.8 months (95% CI, 20.0–33.6 months) in group A and 40.0 months (95% CI, 21.6–58.4 months) in group B. Median OS 2nd was 15.2 months (95% CI, 10.8–19.7 months) in group A and 22.3 months (95% CI, 10.0–34.7 months) in group B.

Metastases resection and treatment group were significantly associated with OS 1nd as well as OS 2nd in both univariate and multivariate analysis, except that metastases resection failed to show prognostic significance for OS 2nd in multivariate analysis (Tables 5, 6).

**Table 5:** Univariate and multivariate analysis for factors associated with OS 1nd.

Variables	Univariate analysis			Multivariate analysis		
	HR	95%CI	*p* value	HR	95%CI	*p* value
**Age at diagnosis as stage IV (years)**						
< 52	1					
≥ 52	0.744	(0.470–1.178)	0.207			
**Gender**						
Male	1					
Female	1.475	(0.912–2.387)	0.113			
**WHO PS**						
0–1	1					
≥ 2	1.013	(0.953–1.076)	0.686			
Tumor histological grade			0.105			
**Localization of the primary tumor**						
Colon	1					
Rectum	0.767	(0.479–1.229)	0.271			
**Number of metastatic sites**						
1	1					
> 1	1. 263	(0.796–2.004)	0.321			
**CEA**						
< 19.99	1					
≥ 19.99	1.088	(0.680–1.740)	0.724			
**Primary tumor resection**						
Yes	1					
No	1.058	(0.662–1.690)	0.813			
**Metastases resection**						
Yes	1			1		
No	1.932	(1.153–3.239)	**0.012**	1.732	(1.026–2.924)	**0.040**
**Chemotherapy used in first-line**			0.432			
Oxaliplatin-based	1					
Irinotecan-based	0.727	(0.436–1.210)	0.219			
Others	1.092	(0.429–2.780)	0.853			
**Chemotherapy used in second-line**			0.211			
Oxaliplatin-based	1					
Irinotecan-based	1.498	(0.934–2.405)	0.094			
Others	0.920	(0.321–2.631)	0.876			
**Treatment group**						
Group A	1			1		
Group B	0.543	(0.338–0.873)	**0.012**	0.605	(0.373–0.980)	**0.041**

Treatment group A: bevacizumab-containing regimens in both first-line and second-line therapies.

Treatment group B: first-line cetuximab-containing regimens followed by second-line bevacizumab-containing regimens.

Bold values indicate significant differences between two groups.

**Table 6:** Univariate and multivariate analysis for factors associated with OS 2nd.

Variables	Univariate analysis			Multivariate analysis		
	HR	95%CI	*p* value	HR	95%CI	*p* value
**Age at diagnosis as stage IV (years)**						
< 52	1					
≥ 52	0.755	(0.477–1.197)	0.232			
**Gender**						
Male	1					
Female	1.503	(0.929–2.432)	**0.097**			
**WHO PS**						
0–1	1					
≥ 2	1.012	(0.952–1.075)	0.708			
Tumor histological grade			0.160			
**Localization of the primary tumor**						
Colon	1					
Rectum	0.824	(0.519–1.310)	0.413			
**Number of metastatic sites**						
1	1					
>1	1.091	(0.688–1.729)	0.711			
**CEA**						
< 19.99	1					
≥ 19.99	1.124	(0.702–1.798)	0.627			
**Primary tumor resection**						
Yes	1					
No	1.066	(0.670–1.697)	0.788			
**Metastases resection**						
Yes	1			1		
No	1.656	(1.006–2.726)	**0.047**	1.486	(0.898–2.459)	0.124
**Chemotherapy used in first-line**			0.467			
Oxaliplatin-based	1					
Irinotecan-based	0.738	(0.445–1.226)	0.241			
Others	1.085	(0.430–2.738)	0.863			
**Chemotherapy used in second-line**			0.253			
Oxaliplatin-based	1					
Irinotecan-based	1.431	(0.892–2.295)	0.137			
Others	0.827	(0.290–2.354)	0.722			
**Treatment group**						
Group A	1			1		
Group B	0.524	(0.328–0.835)	**0.007**	0.561	(0.350–0.900)	**0.016**

Treatment group A: bevacizumab-containing regimens in both first-line and second-line therapies.

Treatment group B: first-line cetuximab-containing regimens followed by second-line bevacizumab-containing regimens.

Bold values indicate significant differences between two groups.

## Discussion

To further confirm the optimal sequence of EGFR and VEGF inhibitors in RAS wild-type left-sided mCRC and explore the influence of first-line biologic agents on second-line ones, we carried out this study and found that patients treated with first-line cetuximab-containing and second-line bevacizumab-containing regimens had better PFS 2nd, OS 1nd and OS 2nd compared to patients with continuation of bevacizumab-containing crossover therapy, and that previous cetuximab use had a promoting effect on the activity of subsequent bevacizumab.

Patients with RAS mutant-type or right-sided mCRC cannot benefit from cetuximab, and only the patients with both RAS wild-type and left-sided mCRC are candidates for anti-EGFR therapy, while bevacizumab shows efficacy regardless of the tumor location or RAS mutation status^8,17–20^. Several important clinical trials and some retrospective studies have shown that first-line EGFR inhibitors exhibited comparable PFS and superior OS compared with VEGF inhibitors in RAS wild-type left-sided mCRC^9,10,21^. In accordance with previous studies, we also found no statistical difference in PFS 1nd between first-line cetuximab- and bevacizumab-containing groups. However, a prolonged OS 1nd was observed in cetuximab group in both univariate and multivariate analysis.

In our study, PFS2nd was significantly prolonged in cetuximab-pretreated patients compared with bevacizumab-pretreated cases, and this observation transformed into a prolonged OS2nd. Improved OS by second-line use of VEGF inhibitors has also been observed in several clinical trials^22–24^. The possible mechanisms have also been demonstrated in several experimental researches. Long-term treatment of CRC cells with EGFR inhibitors induced the emergence of EGFR inhibitor-resistant cells. The acquired resistance to anti-EGFR antibodies emerged at least in part by the selection of cancer cell subpopulations with increased angiogenic potential, as a 5–10-fold increase in the expression of VEGF was observed. Besides, the EGFR inhibitor-resistant cells were more sensitive to anti-VEGF agents both in vitro and in vivo^25,26^. Based on above researches, we may infer that anti-EGFR antibodies induced up-regulation of VEGF at least partly contributed to the better PFS and OS for second-line anti-VEGF agents.

Currently, there are three targeted therapeutic strategies in RAS wild-type mCRC: First-line chemotherapy plus anti-EGFR agents and second-line chemotherapy plus anti-VEGF agents; first-line chemotherapy combined with anti-VEGF agents followed by second-line chemotherapy combined with anti-EGFR agents; and VEGF inhibitor-containing regimens in both first- and second-line treatments. The first strategy has been proved to show advantage over the second one in prolonging OS 1nd, PFS 2nd and OS 2nd^9,10,27,28^. When comparing the latter two strategies, several researches about the second-line choice after progression of first-line treatment with bevacizumab in mCRC patients has been conducted recently. They found that bevacizumab plus standard chemotherapy was superior than that of cetuximab combined with chemotherapy in second-line therapy because the former had longer PFS 2nd and OS 2nd, although some of the differences were not statistically significant^14–16^. Based on the above clinical data and the perception that continuation of anti-EGFR antibodies in second-line is usually not recommended due to low effectiveness, second-line VEGF inhibitor following first-line EGFR inhibitor when disease progresses might be the most appropriate treatment strategy for RAS wild-type left-sided mCRC patients.

There were several limitations in our study. First, as a retrospective study, it was less valuable and convincing due to purely observational nature when compared with prospective studies. Second, only 50 patients in group A and 51 patients in group B were included, and the very finite sample size made the statistical results not accurate enough to draw an undisputed conclusion. In addition, the targeted therapies were in combination with chemotherapy and local treatments (primary tumor or metastases resection) in our study, which might also affect the outcomes. Nonetheless, we have enrolled patients strictly according to the criteria and tried our effort to balance all the clinicopathological factors between two groups to ensure the accuracy of the results.

## Conclusion

Taken together, we further compared the efficacy between first-line cetuximab and bevacizumab in RAS wild-type left-sided mCRC patients with our data, as well as analyzed the influence of first-line anti-EGFR/VEGF agents on second line VEGF inhibitors. Based on our results and the previously reported clinical data, first-line application of anti-EGFR agents provides a favorable condition for promoting the effect of subsequent anti-VEGF agents, and first-line anti-EGFR containing regimens followed by second-line anti-VEGF containing regimens might be the optimal medical strategy for the patients of RAS wild-type left-sided mCRC.

## Methods

### Patients and study design

**Figure 2:**
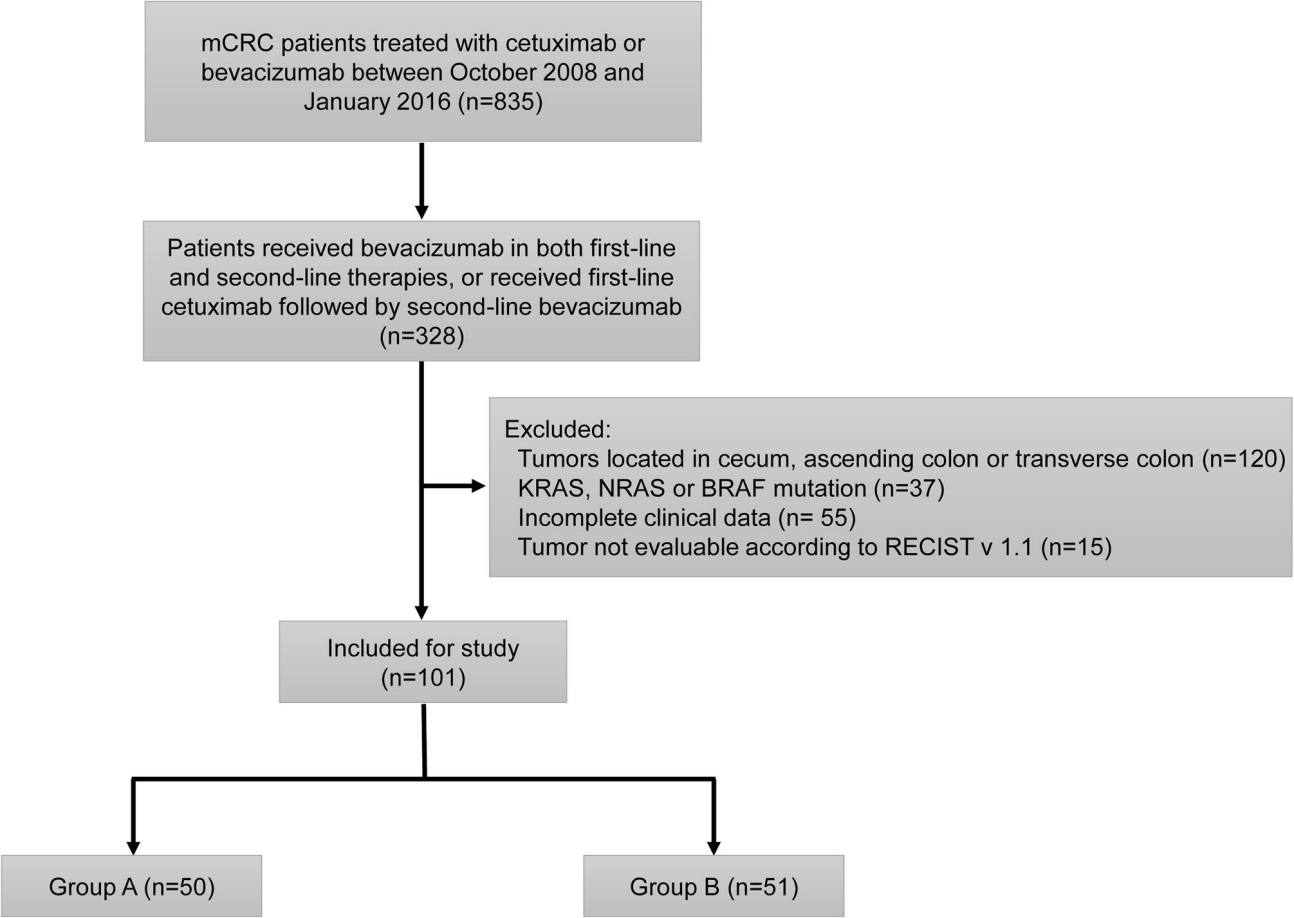
Flow chart depicting patient deposition. mCRC, metastatic colorectal cancer.

In this retrospective study, 101 left-sided mCRC patients with KRAS, NRAS and BRAF wild-type status treated at Sun Yat-Sen University Cancer Center in China from October 2008 to January 2016 were included. Among all these patients, 50 cases received bevacizumab-containing regimens in both first-line and second-line therapies (Group A) and 51 cases received first-line cetuximab plus chemotherapy followed by second-line bevacizumab plus chemotherapy (Group B). Only patients with measurable lesions and underwent tumor assessments during the treatment every 6–8 weeks according to RECIST version 1.1 were eligible. The screening process of enrolled patients is summarized in Fig. 2. Clinicopathological characteristics of the patients are summarized in Table 1.

### Definitions

First-line therapy was defined as administration of bevacizumab- or cetuximab-containing regimens for first time after diagnosis of stage IV disease, and second-line therapy was defined as the start of administration of any anti-cancer drugs from disease progression no matter any changes in regimens. Overall response rate (ORR) meant the proportion of patients achieving complete or partial response according to RECIST version 1.1. Disease control rate (DCR) was defined as the proportion of patients who reached stable disease, partial or complete response according to RECIST version 1.1. PFS 1nd was measured from the beginning of first-line therapy to first disease progression, and PFS 2nd was calculated from the date when second-line therapy started to second progression in disease. OS 1nd referred to the time from first application of first-line therapy to death due to cancer, and OS 2nd was defined as the time from beginning of second-line therapy to death resulting from cancer.

### Statistical analysis

Statistical analyses were performed using SPSS 22.0 software (SPSS Inc, USA). Categorical characteristics, ORR and DCR between two treatment groups were compared using the Pearson Chi square test. Survival probabilities including PFS 1nd, PFS2nd, OS1nd and OS2nd were estimated using the Kaplan–Meier method and survival curves were compared by log-rank test. A multivariate Cox regression model was used to estimate the effects of treatment strategies and other factors on PFS and OS. Only variables with *p* value of less than 0.1 in the univariate model were included for further analysis in the multivariate Cox model. A *p* value of less than 0.05 was considered statistically significant.

### Ethic approval and consent to participate

All methods were carried out in accordance with relevant guidelines and regulations. All experimental protocols were approved by the Research Ethics Committee of Sun Yat-sen University. Informed consent was obtained from all individual participants included in the study.

## Supplementary information

Supplementary information

## Data Availability

All authors had access to the primary data.

## Abbreviations

mCRC: Metastatic colorectal cancer
EGFR: Epidermal growth factor receptor
VEGF: Vascular endothelial growth factor
CR: Complete response
PR: Partial response
SD: Stable disease
PD: Progression of disease
ORR: Overall response rate
DCR: Disease control rate
PFS: Progression-free survival
PFS 1nd: From the beginning of first-line therapy to first disease progression
PFS 2nd: From the date when second-line therapy started to second progression in disease
OS: Overall survival
OS 1nd: From first application of first-line therapy to death resulting from mCRC
OS 2nd: From beginning of second-line therapy to death resulting from mCRC
HR: Hazard ratio
