# Supplementary material for: First-line cetuximab improves the efficacy of subsequent bevacizumab for RAS wild-type left-sided metastatic colorectal cancer: an observational retrospective study
Source: Sci Rep. 2020 Jul 23;10:12336. doi: 10.1038/s41598-020-69230-5 (PMC7378223; doi:10.1038/s41598-020-69230-5)
Supplement: Supplementary file 1 — Supplementary information [file 41598_2020_69230_MOESM1_ESM.docx]

**First-line** **cetuximab improves the efficacy of subsequent** **bevacizumab for** **RAS wild-type** **left-sided** **metastatic colorectal cancer: An observational retrospective study**

Shousheng Liu†^1,2^, Chang Jiang †^1,2^, Lin Yang †^3^, Jinsheng Huang^1,2^, Roujun Peng^1,2^, Xiaopai Wang^4^, Wenzhuo He^1,2^, Long Bai^1,2^, Yixin Zhou^1,2^, Bei Zhang *^1,2^, Liangping Xia *^1, 2^

**Table S1**. Details of third-line treatment for mCRC patients

|  | Number of cases (%) |
| --- | --- |
| Regorafenib | 10 (9.9) |
| FOLFIRI+Bevacizumab | 7 (6.9) |
| Famitinib | 6 (5.9) |
| Irinotecan+Cetuximab | 5 (5.0) |
| FOLFIRI | 5 (5.0) |
| FOLFOX | 4 (4.0) |
| Cetuximab | 4 (4.0) |
| XELOX | 3 (3.0) |
| FOLFOX+Bevacizumab | 3 (3.0) |
| Best supportive care | 3 (3.0) |
| TAS-102 | 2 (2.0) |
| Ziv-aflibercept | 2 (2.0) |
| FOLFIRI+Cetuximab | 2 (2.0) |
| FOLFOX+Cetuximab | 2 (2.0) |
| Xeloda+Bevacizumab | 2 (2.0) |
| S1 | 2 (2.0) |
| Irinotecan | 1 (1.0) |
| PD-1 | 1 (1.0) |
| Apatinib | 1 (1.0) |
| Irinotecan+Bevacizumab | 1 (1.0) |
| S1+Bevacizumab | 1 (1.0) |
| SOX | 1 (1.0) |
| Fruquintinib | 1 (1.0) |
| Xeloda | 1 (1.0) |
| Unknown | 31 (30.7) |
